# Colorectal Cancer Screening in Castilla La Mancha, Spain: The Influence of Social, Economic, Demographic and Geographic Factors

**DOI:** 10.1007/s10900-022-01071-x

**Published:** 2022-02-06

**Authors:** Laura Valiente González, Francisco Escribano Sotos, Ricardo de Miguel Ibáñez

**Affiliations:** 1 Department of Gastroenterology, Virgen de La Luz Hospital, C/Colón, CP: 16002 Cuenca, Spain; 2 University of Castilla, La Mancha, Albacete, Spain; 3 Department of General and Digestive Surgery, Virgen de La Luz Hospital, Cuenca, Spain

**Keywords:** Colorectal cancer, Screening, Rural, Urban, Income, Unemployment

## Abstract

Colorectal cancer (CRC) is a health problem with a significant social impact, accounting for 700,000 deaths a year globally**.** CRC survival rates are increasing as a result of early detection and improvements in society and labor conditions. Differences in CRC have been found depending on place of residence (urban or rural), socioeconomic situation and unemployment, although studies in this regard are limited. The aims of the present study were to determine whether differences exist in diagnostic delay according to place of residence, to analyze the association between socioeconomic level and colonoscopy results and to evaluate CRC risk according to place of residence, income level and unemployment. Retrospective, descriptive and observational study based on colonoscopies performed between May 2015 and November 2018, analyzing relationships between colonoscopy findings of a population screening program and various socioeconomic and demographic variables included in the study (sex, age, place of residence, average annual income, unemployment rate, etc.), and determining any association between such factors and related increases in adenocarcinoma risk. A total of 1422 patients were included in the study. The difference in participation according to sex was greater in rural population (63,4% men/36,6% women in rural areas, 58% men/42% women in urban areas). The mean delayed diagnosis was 59,26 days in both groups. Adenocarcinoma risk was 1.216 times higher in rural population. High-grade dysplasic lesions and adenocarcinoma were more common in municipalities with income < 9000€. However, advanced stage adenocarcinoma was higher in municipalities with income > 9000€. Adenocarcinoma risk was 1,088 times higher in municipalities with an unemployment rate of > 10%. Living in rural areas is not a barrier to access to health care, with no disadvantages identified regarding diagnosis and treatment, thanks to public health policies and the large number of small municipalities near the referral hospital in Cuenca.

## Introduction

Cancer is a public health problem with a significant social impact [1, 2]. CRC is the third most common cancer and the second most deadly worldwide, accounting for 700,000 deaths per year [3]. Survival rates have increased in recent years since population screening programs were initiated and consolidated, although prognosis continues to be associated with age at diagnosis, tumor stage and treatment [4–6]. The primary risk factor for colorectal cancer is age, with 90% of cases occurring in adults aged 50 or over [6].

The increased life expectancy with cancer can be explained by improvements in social and working conditions, early detection, higher levels of education and greater media information. This increase in life expectancy has led to a rise in neoplasm risk [7].

The foremost CRC prognostic factor is the tumor stage at diagnosis, which may be influenced by delayed diagnosis and treatment. Delay can be classified as patient delay (period from symptom recognition to first consultation), primary care delay (time between first consultation and referral to secondary care) and system delay (time from referral to diagnosis). The last two reasons are typically called provider delay. Studies have shown that individuals living in more disadvantaged locations (rural environments) have more limited access to the healthcare system and thus worse outcomes in various diseases [8]. These difficulties have also been observed during the current COVID-19 pandemic [9, 10].

CRC is appropriate for screening programs as it is a major public health problem for which there are early detection tests, thus enabling more effective treatment. Nonetheless, participation in these programs is lower than in other cancer prevention programs (breast, cervix), for various reasons, including the perception that these tests are painful and complex to perform [11].

Polyps are preoneoplastic lesions associated with genetic and environmental factors. Although not all polyps are malignant, 80–90% of those resected in screening programs are adenomatous. Most of such polyps are precursors for colorectal carcinomas, progressing towards severe dysplasia and invasive carcinomas after evolving over approximately 10 years [12].

In Spain, more than 15,000 people die from CCR per year, representing 2.4% of deaths worldwide. Reported incidence rates vary greatly across regions, with no clear reason for these differences. Genetic factors play a significant role, while environmental factors also appear to have an impact on the appearance of neoplasia [13].

Castilla La Mancha is a region in central Spain with a population of approximately 2 million inhabitants. In 2018, of the total CRC cases in Spain, 4.19% were diagnosed in Castilla La Mancha (600 cases), where there were 145 deaths from this cause (3.74% of the national total) [14].

Cuenca is a small province of Castilla La Mancha with 197,222 inhabitants, of which 41,780 are between 50 and 70 years old. It is made up of 238 municipalities distributed over 17,141km^2^ with a single referral hospital (Hospital Virgen de la Luz) that covers medical care from almost the entire province. In 2019, according to statistical data recorded in Cuenca, the incidence rate of CRC, in people between 45 and 70 years old was 69 cases per 100,000 inhabitants, with a mortality rate of 17 per 100,000 inhabitants [15].

International studies have shown clear differences in cancer incidence and mortality, depending on patients’ socioeconomic situation, whether they live in urban or rural areas and the ease of access to treatment [16–18].

An association has been found between socioeconomic level and survival in certain types of cancer [17–19]. These differences are related, at least partially, to a more advanced tumor stage at diagnosis and poorer access to optimal treatment in persons with a low socioeconomic status, which is more pronounced in countries without universal healthcare [20].

These geographical patterns in disease maps might suggest the involvement of risks factors related to ethnic, healthcare and living conditions, as well as the quality of the environment [16].

The characteristics of rural populations are different to those of urban areas, meaning it is important to determine the spatial distribution of the disease with a view to implementing preventive strategies. Furthermore, social determinants, such as economic level and patterns of consumption, have a significant impact on colorectal cancer.

Few studies in Spain have associated cancer incidence with unemployment, but analyses published in different countries have shown direct relationships between adverse socioeconomic conditions and cancer risk. This is arguably related to disadvantaged people presenting less healthy lifestyles (consumption of alcohol and tobacco, obesity, physical inactivity and lower consumption of fruit and vegetables) [21].

In addition, income significantly affects access to programs to detect, treat and control cancer, especially in countries where health coverage is not universal. Poverty is related to adverse scenarios of cancer detection and affects the management and control of pathologies. The impact of the disease also appears to be associated with education level and cultural background [22].

Regardless of individual factors, certain contextual factors in the geographical area explain the standard of health, such as the environment, urban development, leisure facilities, service provision, etc. Additionally, despite the impossibility of accessing individualized data in some situations, the geographical representation of health indicators arguably suggests spatial patterns that otherwise might not be detected, which can be useful to formulate etiological hypotheses and to guide epidemiological research. Geographical patterns identified for certain tumors in studies on small areas of mortality in Spain suggest that, territorially, environmental factors have a substantial effect on tumor etiology [23].

The aims of the present study were as follows:

- To determine the existence of differences in delayed diagnosis according to place of origin (urban/rural), distance to health center and referral hospital.
- To evaluate the effect of delay on colonoscopy findings and tumor stage at diagnosis.
- To analyze the association between socioeconomic status on participation in a colorectal cancer screening program and the findings of colonoscopies performed as part of the same program.
- To determine whether place of residence, annual income and the unemployment rate in the municipality of residence are risk factors for colorectal cancer.

## Methods

### Population and Sample

The Integrated Healthcare Service of Cuenca comprises 34 sectors with the Virgen de la Luz Hospital, located in the provincial capital as the only referral hospital [24].

The Population Screening Program for CRC in the Autonomous Community of Castilla La Mancha began as a pilot program in three hospitals in April 2015 [25], and was extended to the whole region in 2016. It is aimed at men and women aged between 50 and 69, resident in the region, without previous colorectal pathology and who have not undergone a colonoscopy in the last five years.

The program was rolled out in Cuenca in 2015 and continues today. It is free of charge, with participants being invited to have a fecal occult blood test (FOBT) once every two years in their primary care center, and, if positive, a subsequent colonoscopy is performed in the referral hospital.

The study population comprised all individuals in the province of Cuenca that had undergone a colonoscopy at the Virgen de la Luz Hospital, the only referral hospital in the Cuenca Healthcare Area, following a positive FOBT test conducted as part of the population screening program for colorectal cancer between May 2015 (start date of the CRC population screening program in Castilla La Mancha) and November 2018 (end date of the first round of the CRC population screening program in Castilla La Mancha). The cases were selected by systematic inclusion. The exclusion criteria drew on those established in the population screening program. These were as follows: personal history of CRC, adenomas or inflammatory bowel disease, prior colectomy, family history of CRC, colonoscopy performed in the last five years, physical or mental incapacity, change in autonomous community of residence, or exitus. We collected sociodemographic and economic data (age, sex, place of residence, distance from health center and referral hospital, annual income per person/year, unemployment rate) and clinical data (colonoscopy findings, presence of adenomas and adenocarcinomas, complications during the procedure, treatment received).

The data on population, census, annual income and unemployment rate per municipality were collected from records at the Cuenca Provincial Council and via institutional platforms. The study was conducted using population from 2016–2017.

The delay between the FOBT and colonoscopy was calculated according to the difference between the dates the fecal sample was collected and the first colonoscopy was performed, with the latter being taken as the first contact with the specialized care service.

The FOBT results were obtained from provincial records provided by the management of the Virgen de la Luz Hospital. The colonoscopy findings and treatment data were collected from anonymized data compiled in clinical history records stored in Mambrino XXI (health data management software).

According to their histology, and following the revised Vienna classification, polyps were classified into non-neoplasm, low-grade mucinous neoplasm, high-grade mucinous neoplasm, and carcinoma. Adenocarcinomas were classified by stage, following the TNM cancer staging classification designed by the American Joint Committee of Cancer. The qualitative variables were presented using absolute and relative frequencies and the quantitative variables were based on means and confidence intervals.

The patients were divided into two groups according to the number of inhabitants in their municipality of residence: rural if < 10.000 inhabitants, and urban if > 10.000 inhabitants. They were also divided into three groups depending on distance from the nearest health center (< 10, 10–20, > 20 km) and into a further three groups, depending on distance to the hospital (< 10, 10–50, > 50 km).

Two groups were created according to annual income per person (< 9000€ and > 9.000€). The unemployment rate was calculated based on the number of individuals aged between 50 and 69 years in relation to the census population of their municipality in this age group. The patients were thus divided into two groups (< 10%, > 10%).

## Data Analysis

The study design is a retrospective, descriptive, analytical and observational. It was conducted in the Gastroenterology Department of the Virgen de la Luz Hospital in Cuenca. The data were processed using Microsoft Excel 2016 and analyzed using SPSS version 22.

We conducted a descriptive analysis of the data from the colonoscopy reports and from the follow-up and treatment period. Sample normality was assessed using the Kolmogorov–Smirnov test. The analysis was performed according to age, sex, place of residence (rural/urban), etc., using cross tabulation analysis, evaluating whether variables were correlated using the Mann Whitney U test for the quantitative variables and Pearson’s chi-square test for the qualitative variables.

An explanatory binary logistic regression model was used to estimate the relationship between adenocarcinoma detected during colonoscopy and the sociodemographic variables. Presence of adenocarcinoma was included as the dependent variable and place of residence as the independent variable.

As regards delayed diagnosis, we considered the mean delay depending on place of residence and, using contingency tables, we examined the correlation between delay and colonoscopy findings, presence of adenocarcinoma and tumor stage at diagnosis.

We studied the mortality rate associated with various factors (distance to health center and hospital, income and unemployment rate) using Cox regression analysis. To avoid competitive risk bias, all causes of death other than CRC were excluded. Statistical significance was set at *p* < 0.05.

## Results

A total of 1422 patients from 166 municipalities in the province of Cuenca were included in the study.

**Fig. 1:**
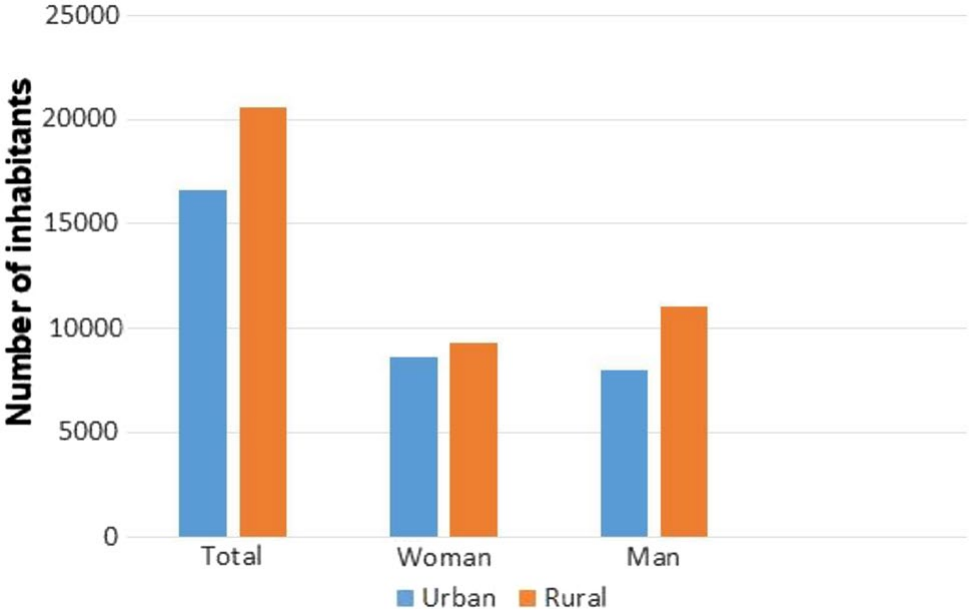
Census population aged between 50 and 69 years in rural and urban municipalties

Figure 1 shows the census population in rural and urban environments aged between 50 and 69 years. According to the census population data, 54% of individuals in rural areas were men and 46% were women, while, in urban areas, 48% were men and 52% were women. The study population comprised 61.3% men and 28.7% women. Mean age was 60.15 years (CI 59.83–60.46), 60.37 years in rural areas and 59.8 years in urban areas. Of the colonoscopies performed, 57.6% corresponded to rural population and 42.4% to urban population.

The differences in participation according to sex was greater in the rural population, with this difference being statistically significant (*p* = 0.043). In rural environments, 63.4% of patients were men and 36.6% women, while the percentages in urban areas were more similar, 58% men and 42% women.

Table 1 shows the sociodemographic, histopathological and clinical characteristics of the sample.

**Table 1:** Sociodemographic, histopathological and clinical characteristics

	**Rural** *n* (%)		**Urban** *n* (%)	
	**Women**	**Men**	**Women**	**Men**
	304 (36.6)	527 (63.4)	256 (42)	356 (58)
**Complete colonoscopy**	283 (93.1)	501 (95)	234 (91.4)	338 (95)
**Incomplete colonoscopy**	21 (6.9)	26 (5)	22 (8.6)	18 (5)
**Sedation**				
No sedation	2 (0.7)	2 (0.4)	1 (0.4)	2 (0.6)
Midazolam + fentanyl	222 (73.1)	360 (68.3)	143 (55.9)	225 (63.2)
Propofol	76 (25.2)	158 (30)	89 (34.7)	114 (32)
Anesthetist	4 (1)	7 (1.3)	23 (9)	15 (4.2)
**Colonoscopy findings**				
Normal	142 (46.7)	126 (24)	124 (48.4)	83 (23.3)
Lesions	162 (53.3)	400 (76)	131 (51.6)	273 (76.7)
**Histopathology**				
No lesions/ Hyperplastic polyps	167 (54.9)	172 (32.7)	120 (58.6)	114 (32.1)
Low-grade adenoma	110 (36.2)	281 (53.3)	85 (33.2)	198 (55.6)
High-grade adenoma	17 (5.6)	37 (7)	9 (3.5)	19 (5.3)
Adenocarcinoma	10 (3.3)	37 (7)	12 (4.7)	25 (7)
**Tumor stage at diagnosis**				
No tumor	294 (96.65)	490 (93.1)	244 (95.2)	332 (93.25)
Stage 0	6 (2)	11 (2.1)	5 (2)	8 (2.2)
Stage I	3 (1)	5 (0.9)	2 (0.8)	6 (1.7)
Stage II	0	7 (1.3)	2 (0.8)	3 (0.85)
Stage III	1 (0.35)	8 (1.5)	3 (1.2)	5 (1.4)
Stage IV	0	6 (1.1)	0	2 (0.6)
**Other lesions**				
Inflammatory bowel disease	2 (0.7)	7 (1.3)	1 (0.4)	2 (0.6)
Submucous lesion	2 (0.7)	4 (0.8)	5 (2)	4 (1.2)
Angiodysplasias	1 (0.35)	7 (1.3)	1 (0.4)	4 (1.2)
Ulcer	1 (0.35)	3 (0.6)	0	2 (0.6)
Diverticles	61 (20.1)	114 (21.6)	50 (19.5)	91 (25.6)
Hemorrhoids	100 (32.9)	148 (28.1)	89 (34.8)	120 (33.7)
**Complications**				
Immediate hemorrhage	8 (2.6)	16 (3)	2 (0.8)	13 (3.7)
Delayed hemorrhage	1 (0.35)	2 (0.4)	1 (0.4)	6 (1.7)
Vasovagal reaction	0	2 (0.4)	0	1 (0.3)
Desaturation	1 (0.35)	0	0	0
Perforation	0	1 (0.2)	1 (0.4)	1 (0.3)

The mean diagnostic delay was 59.26 days (CI 55.40–63.13) for both groups. According to sex, the delay in the case of men was 58.8 days (CI 95% 54.02–64.05) and in women 60.01 days (IC 95% 54.63–66.99), with this difference showing no statistical significance (p 0.758).

Of the 166 municipalities, 71.7% had a health center, while, in 28.3%, patients had to travel to another municipality for the FOBT. Differences in the delay were found between individuals from municipalities with a health center and those where the nearest health center was located at > 20 km, with the delay times being 58.1 days and 73.8 days, respectively (*p* = 0.159).

The colonoscopy findings showed similar percentages of lesions in patients from rural and urban areas (32.3% and 33.8%). We estimated the relationship between presence of adenocarcinoma and place of residence, using binary logistic regression (BLR). We found adenocarcinoma risk was 1.216 (CI 95%:0.648–2.280) times higher in rural population. The number of lesions was higher in residents of municipalities located at > 50 km from the hospital, among whom, 2% more high-grade lesions or adenocarcinomas were diagnosed, with these data being statistically significant at 90% (*p* = 0.089).

The number of infiltrative tumors was higher in persons from municipalities located at < 50 km from the hospital (*p* = 0.581). As regards tumor stage, 32.6% of the tumors diagnosed in individuals living < 50 km from the hospital were advanced (Stages III–IV), while in those from municipalities at > 50 km, we found 27.07% of the tumors were at an advanced stage.

We analyzed the difference between sexes in the number of normal colonoscopies, with the difference being statistically significant (*p* < 0.001) in favor of women in both urban and rural areas. We estimated the relationship between presence of colonoscopy findings and sex, which revealed a significantly lower risk, 0.342 (CI 95%: 0.273–0.429) in women (*p* < 0.001).

The relationship between presence of adenomas and adenocarcinomas in the colonoscopy was statistically significant (*p* = 0.003) for men from urban areas, but not for those in rural areas. As regards lesion stage and sex, we found a statistically significant (*p* = 0.026) relationship between advanced stage tumors and men in rural municipalities but not in urban municipalities.

High-grade dysplastic lesions and adenocarcinomas were more common in residents in municipalities with an average income < 9000€ (21.6% vs 16.3%). Our BLR analysis on the relationship between adenocarcinoma and income showed that risk in municipalities with income < 9000€ was 1.051 (CI 95%: 0.673–1.649) times higher than in municipalities with an average income > 9000€ (*p* = 0.827). However, diagnosis of advanced stage adenocarcinomas was higher in municipalities with income > 9000€/year, 34.4% vs 22.5% (*p* = 0.277).

Adenocarcinoma risk associated with unemployment rate was 1.088 (CI 95%: 0.790–1.497) times higher in municipalities with an unemployment rate of > 10%, according to the BLR (*p* = 0.606) (Table 2).

**Table 2:** Characterization of lesions (adenoma/adenocarcinoma) according to sociodemographic variables

	**High-grade adenomas *n* (%)**	***Adenocarcinoma *** ***n (%)***
**Place of residence**		
Rural	54(66)	47(56)
Urban	28(34)	37(44)
**Sex**		
Woman	26(32)	22(26)
Man	56(68)	62(74)
**Unemployment rate municipality**		
< 10%	20(24)	13(15)
> 10%	62(56)	71(85)
**Income per person/year municipality**		
< 9000€	43(52)	34(40)
> 9000€	39(48)	50(60)

During the study period, 7 CRC deaths were recorded. Of these patients, six were men and one was a woman; five lived in rural areas (four men and one woman) and two in urban areas.

A Cox proportional risk analysis was conducted, finding an increased mortality rate was associated with living in a rural area, annual income/per person > 9000€ and unemployment rate > 10%, with the risk related to unemployment rate being statistically significant (*p* = 0.006).

## Discussion

CRC is the most prevalent cancer in Spain and the second deadliest across all the country’s provinces [26]. The CRC incidence and mortality in Cuenca in 2018 were higher than the national average [14].

Few studies have addressed the differences between rural and urban population in CRC screening programs at either national or international level. In contrast to the findings of previous studies, we found higher participation in rural areas [2, 25–27]. This might be due to the considerable number of municipalities with > 10.000 inhabitants in the Cuenca healthcare area.

We found the number of colonoscopies performed was lower in women from both urban and rural areas, with the difference being more notable in rural settings, which is inconsistent with the findings of previous studies [2].

No difference in provider delay was found between rural and urban areas, which is again counter to previously published results [8, 13, 28]. Differences were detected, however, between patients living in locations with a health center and those living > 20 km from the nearest health center. Longer delay was not found to be related to distance from the hospital, which coincides with the findings of a study conducted in the United States [28], but is inconsistent with the results reported in other research [29]. Although provider delay showed no differences related to the distance from the hospital, differences were found as regards colonoscopy findings, where a higher percentage of lesions was found in patients that lived > 50 km from the hospital, while the percentage of advanced stage tumors was also slightly higher in this group.

As reported in previous studies, being male is a significant risk factor for CRC [2, 16, 30, 32]. Our study found a higher percentage of adenocarcinoma in men in both the rural and the urban group, in contrast to the findings of other studies [31, 33]. This may be because the population profile is different in the province of Cuenca from that of municipalities in other studies.

We also found that place of residence, annual income and unemployment rate were positively associated with the presence of adenoma and adenocarcinoma, which is consistent with previous research [30]. However, we also found that, at diagnosis, the tumor stage was more advanced in residents of municipalities with a higher average income.

The analysis also revealed a higher CRC mortality risk in populations with lower income and higher unemployment. The association with unemployment rates, as suggested by previous studies, might be due to less healthy lifestyles in unemployed individuals (obesity, sedentary lifestyle, increased tobacco consumption) [21].

## Limitations

We were unable to access individuals’ socioeconomic data (annual income and unemployment), using, instead, population data.

Data were not available for the smallest municipalities and we thus had to use mean provincial data, and some individuals included in the study might have been registered in locations that were not their usual place of residence.

As the analysis was conducted only on the population in the Cuenca Healthcare Area, it might not be possible to extrapolate the results to provinces with a different geographical distribution.

To conduct the study, we used estimated census, income and unemployment data for 2016 and 2017, as annual records do not exist, while patients whose colonoscopy was performed between 2015 and 2018 were included in the study.

## Conclusions

Our results form the basis for future analyses relating geographical distribution to risk factors for CRC, thus enabling measures to be taken for prevention and early detection.

The main contribution of our study is that, in contrast to previous studies, we found that living in a rural area is not a barrier to access to health care, with no disadvantages identified regarding diagnosis, follow-up and treatment according to place of residence. These findings may be the consequence of public policies in the Autonomous Community of Castilla La Mancha. They might also be the result of the large number of small municipalities located near the referral hospital, which facilitates health measures and counters the negative effect of low population density and the disperse distribution of municipalities on access to health care. Additionally, we found lower female participation in the population screening program, especially in rural areas.

The study reveals an association between income, unemployment and adenocarcinoma diagnosis, which suggests that one of the measures required to improve public health is to provide support so the population can have access to work and decent wages.

Obstacles to healthcare access should be studied in order to promote measures aimed at reducing gender or socioeconomic gaps in participation in screening programs.

## Abbreviations

CRC: Colorectal cancer
FOBT: Fecal occult blood test
BLR: Binary logistic regression
